# Medical Orthodoxy in the Far East

**Published:** 1898-06-04

**Authors:** 


### Medical Orthodoxy in the Far East.

Eastern questions are apt to be difficult of solution, and the last which has come to lightis one of peculiar complexity. It has to do with the relation of orthodox and unorthodox medicine, and, like the questions which occur in relation to high politics, it answers itself in a diametrically opposite manner according to the point of view from which it is regarded. The problem is shortly this: Should the Government support the quacks? Plague has shown itself in Hong Kong, and the Hong Kong Sanitary Board is again at work, but, just as has been the case in India, great difficulties are being encountered in dealing with the natives. In fact, it would appear that the Chinese fear death by plague far less than they do treatment by European methods. They refuse to notify the cases, they object to have their houses "cleaned down," and they throw their dead out into the streets lest the discovery of a plague case should bring upon them the unwelcome attention of the sanitary officials.

Under these circumstances it has been strongly urged upon the authorities that if the Chinese were allowed to clean their own houses they would not feel the same objection that they seem to do at present to the notification of the disease. The Sanitary Board, however, declines to put faith in Chinese methods of cleaning and disinfection, and, notwithstanding the strong representations made by the Acting-Governor as to the evil consequences which are likely to ensue from persisting in a course which tends to put the whole native population in opposition to all sanitary measures, the Board maintains that nothing short of such a thorough cleaning as in former epidemics has been enforced under European supervision, and in some cases has been actually undertaken by European troops, is likely to be effectual in stopping the progress of the disease. As a compromise, however, and as a sop to native prejudice and fanaticism, it has been decided that a hospital shall be opened by the Sanitary Board, under European medical supervision, where patients shall be given the option of being treated by European or by Chinese methods. It is in regard to this question, that is, in regard to the ethics and morality of giving public support to an institution so arranged as to enable the Chinese to physic themselves in their own manner, and to die in their own way, that the discussion centres.

This official recognition of the right of a patient to choose his own treatment is quite opposed to Western conceptions of scientific medicine. Orthodox medicine is, in fact, so wide, and the fact that there is room within the pale for every form of treatment which is true and is founded upon observation, is so obvious that we cannot but regret this granting of public recognition to methods which are known to be both false and foolish; and we quite agree with much that has been put forward by the principal civil medical officer at Hong Kong in regard to our giving official sanction to the administration of absurd nostrums to dying men. Looking at the question from the standpoint of ethics and morality, it is, surely, wrong that a Sanitary Board should hand over to quacks and pretenders the treatment of patients in its own hospital—"optional" hospital, though it be. Ethics and morality, however, are not everything; and we can quite believe that those who advocate these unorthodox proceedings take what are called large views, and care but little about what happens to individual patients so only that the cases get reported and isolated. In this they claim that this proceeding has proved itself successful, the number of plague cases notified in the epidemic of 1894, when this method was adopted, having doubled so soon as the "optional" hospital was opened. Hence the decision to repeat the experiment.

Much, no doubt, is to be excused to those who are face to face with plague; but this is the thin end of a big wedge, and we do not like it. In the abstract the question is the old one on which so much sophistry has been expended in every age: Is it right to do evil that good may come? In the practical work of life, however, such problems have to be solved on their merits, and the form the solution takes depends much upon point of view. Now in a town threatened with plague the point of view is apt to be that of the sanitarian, and the lives of a few Chinamen and the sensibilities of a few sticklers for medical orthodoxy are but feathers in the scale against anything which will promote the isolation of the disease. So does morality hinge upon circumstances.

